# Predictive factors of mortality in patients with COVID-19 in Guinea: analysis of the first 140 cases admitted to intensive care unit

**DOI:** 10.11604/pamj.2021.38.205.27078

**Published:** 2021-02-23

**Authors:** Joseph Donamou, Abdoulaye Touré, Amadou Yalla Camara, Djiki Camara, M’mah Lamine Camara, Abdourhamane Dine Traoré, Mariame Mohamed Camara, Almamy Bangoura, Boubacar Atigou Dramé, Luc Kouessi Sossa, Jean-Marc Roméo Abékan, Axel Irvin Edemessi, Sow Mamadou Saliou

**Affiliations:** 1 Gamal Abdel Nasser University of Conakry, Anaesthesia and Intensive Care Service of the Donka National Hospital Conakry, Conakry, Guinea,; 2 Gamal Abdel Nasser University of Conakry, Anaesthesia and Intensive Care Service, Ignace Deen National Hospital Conakry, Conakry, Guinea,; 3 Gamal Abdel Nasser University of Conakry, Infectious and Tropical Diseases Department of the Donka National Hospital, Conakry, Guinea

**Keywords:** Mortality, factors, COVID, intensive care unit, Guinea

## Abstract

**Introduction:**

the objective was to identify the predictive factors contributing to COVID-related deaths in Intensive Care Unit.

**Methods:**

this was a 4-month (12^th^ March to 12^th^ July 2020) cross sectional study carried out in the intensive care unit of the COVID treatment center of Donka National Hospital, the only hospital with a COVID intensive care unit in Guinea.

**Results:**

during our period of study 140 patients were hospitalized in the COVID intensive care unit and 35 patients died (25%). In univariate analysis, the occurrence of death was associated with: confusional syndrome (p<0.001), time to admission (p<0.001), use of an inotropic or vasopressor (p<0.001), Brescia score ≥ 2 (p=0.004), non-invasive ventilation (p=0.011), stroke (p=0.014), Acute Respiratory Distress Syndrome (ARDS) (p=0.015), male (p=0.021), provenance (p=0.021), acute renal failure (p=0.022), pulmonary embolism (p=0.022), invasive ventilation (p=0.022), and age > 60 years (p=0.047). In multivariate analysis, the factors predictive of mortality were: Acute Respiratory Distress Syndrome (ARDS) (OR= 6.33, 95% CI [1.66-29]; p=0.007), a Brescia score ≥ 2 (OR =5.8, 95% CI [1.7-19.2]; p=0.004) and admission delay (OR =5.6, 95% CI [1.8-17.5]; p=0.003).

**Conclusion:**

our study shows that the acute respiratory distress syndrome, then the Brescia score ≥ 2, and finally the time to admission to intensive care were all associated with an increased risk of death for patients. These results are different from those reported in Asia, Europe and North America.

## Introduction

The acute respiratory syndrome caused by Coronavirus-2 (SARS-CoV-2), originally described in China, and is responsible for the pandemic we now know as Coronavirus disease 19 or COVID-19. The coronavirus epidemic was declared a “global public health emergency” by the World Health Organization (WHO) on 30^th^ January 2020 [1]. Africa was affected later than other continents, but by 3^rd^ May all African countries had reported at least one case. As of 9^th^ June 2020, Africa had nearly 200,000 confirmed cases and more than 5,000 deaths, with 25 countries having more than 1,000 active cases [2]. The pandemic has led to a considerable number of hospitalizations, particularly in intensive care, with many deaths worldwide. Among patients admitted to intensive care, mortality is variable, strongly associated with age, fragility score and prior co-morbidities, but can reach 30% or even 50% [3,4]. Estimating the case-fatality rate at the level of the total population is useful for understanding the average severity of a pandemic, but it is also crucial to identify patients who are at risk of dying within a population during the pandemic. Information on risk relative to different patient characteristics, especially co-morbidities, allows health care providers to focus on the most vulnerable and improve the allocation of health resources to those who need them most [3,4] and have a real chance of survival. Most analyses of mortality factors have only included studies from Asia, Europe and North America, making it difficult to generalize. In addition, few African studies have focused on potential COVID-related risk factors for mortality. The objective of this study was to identify COVID-related mortality factors in intensive care units in Guinea.

## Methods

It was a 4-month (12^th^ March to 12^th^ July 2020) cross sectional study conducted in the Intensive Care Unit of the COVID Treatment Center of Donka National Hospital, the only hospital with a COVID Intensive Care Unit (ICU) in Guinea, a country of 12.4 million inhabitants. This study was approved by the national ethics committee for health research (notice number: 119/CNERS/20). Due to the nature of the observational examination of the study, we waived the need for informed consent from each patient. The data were collected anonymously and used for purely scientific purposes. All patients tested (by RT-PCR) positive for SARS-CoV-2 admitted to the Intensive Care Unit (ICU), who deceased or discharged cured were included. We excluded deaths in the ICU in patients who were suspected but not formally diagnosed with COVID, and patients who died in normal hospitalization, outside the intensive care unit. Laboratory confirmation methods for SARS-CoV-2 were performed at the Pasteur Institute of Guinea using a reverse transcriptase polymerase chain reaction (RT-PCR) test on a nasopharyngeal swab.

**Case and non-case definitions and data collection:** all patients were admitted to the hospital through the triage service. Patients were managed with a specific pharmacological protocol derived from the national COVID patient management protocol. This treatment included systemic corticosteroids, hydroxychloroquine, antiretroviral drugs, low molecular weight anticoagulants, antibiotics and oxygen therapy. All symptoms reported on admission were documented, including COVID symptoms as defined by the Centers for Disease Control (CDC) [5]: fever (subjective or temperature ≥ 38°C), cough, dyspnea, chills, muscle pain, recent loss of taste or smell, vomiting or diarrhea, sore throat. Respiratory distress was assessed at admission by the Brescia score, which evaluates the severity of respiratory disease in COVID patients. It uses clinical criteria to assess non-intubated patients, assigning a score from 0 to 4, with one point for each of the following four criteria: [6] criterion 1: patient is dyspneic or unable to pronounce a complete sentence at rest or during minimal activity; criterion 2: breathing rate > 22 cycles/min; criterion 3: PaO2 < 65 mmHg or SpO2 < 90%; criterion 4: significant worsening of chest imaging (X-ray, computed tomography (CT) scan).

**Data were collected progressively from the patients´ medical records:** the specific comorbidities established on the basis of data on COVID were included: age over 60 years, obesity, hypertension, diabetes, cardiovascular diseases, chronic obstructive pulmonary diseases, chronic liver diseases, cancers and immunosuppression of any origin. Obesity was defined on the basis of a body mass index (BMI) greater than or equal to 30, calculated by dividing weight in kilograms by height in meters squared, for all patients. Routine radiological assessment, i.e. chest X-ray and blood tests were not available at the time of the study. Complications of the disease were also collected. Acute Respiratory Distress Syndrome (ARDS) was diagnosed according to the Kigali definition, which defines ARDS according to the following criteria [7]: appears within one week of a known clinical accident or the onset or aggravation of respiratory symptoms; a SpO_2_/FiO_2_ report ≤ 315; bilateral opacities not fully explained by effusions, lobar/pulmonary collapse or nodules; respiratory failure that cannot be fully explained by heart failure or fluid overload.

The “death” event corresponded to a death during hospitalization in the department during our study period. For patients who met the eligibility criteria, the following groups of variables were collected from the medical record: epidemiological, clinical, therapeutic and evolutive variables were recorded on a standardized data collection form which was an adapted version of the WHO form: “COVID-19 CASE REPORT FORM_RAPID CORE_version 8 April 2020_revised 13 July 2020” [8]. All the variables were collected and then analyzed according to the status of ‘living’ or ‘deceased’ during hospitalization. At the end of the data collection, we analyzed the influence of these variables on mortality. For the main criterion, we investigated whether variables collected on admission were predictive of death during hospitalization.

**Statistical analysis:** the answers to the questionnaire were first coded and then entered using Epi Data version 3.1 software and analyzed using STATA 15 software. The ICU mortality rate and its 95% confidence interval were determined. To investigate the statistical relationship between the different factors and mortality in patients with coronavirus disease in the COVID ICU, we first compared the two groups (dead and alive) using the Chi-square test or the exact Fisher test when the theoretical number of patients was less than 5. Then, a univariate analysis was carried out to determine the possible association of mortality with each factor studied. Finally, multivariate logistic regression analysis was used to analyze the factors associated with mortality. The statistical significance threshold was chosen at p=0.05. The results were presented using means, standard deviations with 95% confidence intervals and Odd Ratio.

## Results

**Mortality rate and presentation of the general population:** during the study period, 140 patients were hospitalized in the intensive care unit, 35 of whom died. Thus, we recorded an intensive care mortality rate of 25%. The socio-demographic characteristics of the 140 patients admitted to ICU COVID-19 are presented in Table 1 and those of the 35 patients who died are shown in Table 2.

**Table 1:** socio-demographic characteristics of the first 140 COVID patients admitted to the ICU of Donka Hospital

Characteristics	Number (n=140)	Percentage (%)
**Sex**		
Male	110	**79**
Female	30	21
**Age in year: mean±SD=58 ±14**		
<30	4	3
30-39	10	7
40-49	21	15
50-59	29	21
>60	76	**54**
**Occupation**		
Informal sector	30	21
Formal sector	95	**68**
Housewife	15	11
**Provenance**		
Rural area	18	13
Urban area	122	**87**
**BMI mean±SD=26±4**		
<30	126	90
≥30	14	10
**Ethnic group**		
African	132	**94**
Others	8	6

The general population was composed of the majority (79%) of our patients were male, the age group >60 years (54%) was the most represented. The civil servants were the most numerous with 68%, the patients 87% came mainly from urban areas

**Table 2:** socio-demographic characteristics of the 35 COVID patients deceased in the ICU of Donka Hospital

Characteristics	Number (n=35)	Percentage (%)
**Sex**		
Male	**31**	**89**
Female	4	11
**Age (years):** m**ean±SD = 64±11**		
40-49	5	14
50-59	6	17
≥ 60	**24**	**69**
**Occupation**		
Formal sector	**27**	**77**
Informal sector	**7**	20
Housewife	1	3
**Provenance**		
Urban area	**34**	**97**
Rural area	**1**	3
**BMI mean±SD**	**26±4**	
<30	29	**83**
≥30	6	17
**Ethnic group**		
African	34	**97**
Others	1	3

Most of the deceased patients were male (89%), 69% were >60 years of age

**Comparison of the socio-demographic and clinical characteristics between the subgroup of recovered versus the subgroup of deceased:** the mean age was 65 years with extremes of 40 years and 82 years in deceased patients and 57 years with extremes of 23 years and 87 years in surviving patients. There was a predominance of the male sex in both subgroups. The time from the onset of the first symptoms to admission to the COVID intensive care unit was longer in the deceased group with a mean of 14 days (extremes: 8 and 20 days), whereas in the survivors group it was 12 days (extremes: 8 and 22 days). The mean Brescia score for the deceased was 3, while for the survivors it was 2. Clinical signs at admission were mainly represented by dyspnea in both groups with a mean respiratory rate of 28 cycles/min in the survivors and 29 cycles/min in the deceased (NS); hypoxemia was found with a mean oxygen saturation of 85% in ambient air with extremes of 39% and 100% in the survivors and a lower oxygen saturation of 75% in ambient air in the deceased with extremes of 30% and 100%.

**Results of analysis by logistic regression**: the occurrence of a death in the COVID intensive care unit was associated in univariate analysis: Confusional syndrome (p < 0.001), time to admission (p < 0.001), use of inotropic or vasopressor therapy (p < 0.001), Brescia score ≥ 2 (p=0.004), non-invasive ventilation (p=0.011), stroke (p=0.014), to Acute Respiratory Distress Syndrome (ARDS) (p=0.015), male (p=0.021), locality (p=0.021), acute renal failure (p=0.022), pulmonary embolism (p=0.022), invasive ventilation (p=0.022), and age >60 years (p=0.047). In multivariate analysis, the factors predictive of mortality during intensive care hospitalization were: admission time (OR = 5.6, 95% CI [1.8-17.5]; p = 0.003), a Brescia score ≥ 2 (OR =5.8, 95% CI [1.7-19.2]; p = 0.004) and ARDS (OR= 6.33, 95% CI [1.66-29]; p = 0.007). The multivariate mortality analyses were summarized in Table 3.

**Table 3:** predictors of mortality among COVID patients admitted to the ICU of Donka Hospital, multivariate analysis (n=140)

Characteristics	Adjusted Odds Ratio (AOR) [95 % IC]	p-value
**Time between first sign and admission**		
<14	Reference	
≥14	6 1**[.77-17.49]**	**0.003**
**Score of Brescia**		
Score < 2	Reference	
Score ≥2	6 **[1.74-19.17]**	**0.004**
**ARDS**		
No	Reference	
Yes	6.33 **[1.72-29.0]**	**0.007**

The predictive factors for death were the presence of ARDS, the Brescia score ≥2 and the time between first signs and admission

## Discussion

This study showed a 25% mortality rate in the COVID ICU. In multivariate analysis, the time of admission to the ICU greater than 14 days from the onset of the first symptoms, the Brescia score greater than or equal to 2 and acute respiratory distress syndrome (ARDS) were found to be predictive of mortality in COVID patients admitted to the ICU. One of the important characteristics of infectious diseases, particularly those caused by a new pathogen such as SARS-CoV-2, is their severity, the ultimate measure of which is their ability to cause death [9]. Early studies of adults with COVID disease admitted to intensive care units (ICUs) have shown high overall mortality: 62% in China [10], 26% in Italy [11], 50% in the United Kingdom [12] and 50-67% in the United States [13], and mortality of more than 80% in patients requiring invasive mechanical ventilation [13]. Mortality was variable in subsequent reports but exceeded 50% in almost half of the published studies [2]. Experts, including WHO experts, predicted a health catastrophe in Africa, given the poor level of the health system in most sub-Saharan African countries compared to that of developed countries, and especially because African-American and African-European populations have paid a heavy price for COVID in developed countries [1,3]. Contrary to the predictions of these experts, the mortality rate observed in African countries was comparable, if not low, to that observed in northern countries with a well-structured health system, well-equipped personnel with technically advanced health equipment and logistics, and more efficient than those in African countries. On the African continent, the overall mortality rates reported are very disparate, ranging from 0% to more than 10% [1,2]. In our study, the ICU mortality rate is lower than in the United States and France, where rates of 50% and 40% respectively were reported [13,14]. It was similar to those observed in Italy and China, where rates of 26% and 26.4% respectively were found [11,12].

The low mortality observed in our study is thought to be due to a number of factors: the mean age of patients admitted to the ICU in our study was lower than that found in studies carried out in Western countries where this rate was very high. Indeed, we now know that advanced age > 60 years is a major mortality factor in patients admitted to the ICU [10,14-16]. Studies carried out in the United States and Europe show that the mean age of patients who died from COVID in the ICU was over 60 years old (64 years old in the USA, 63 years old in Italy and 62.5 years old in France) [11,13,15]. Our study population was younger than those found in western studies. The second hypothesis is the presence of co-morbidities, which represent a major aggravating factor and mortality of the COVID [13,14,17]. In our study, patients rarely had several co-morbidities at the same time. Moreover, these co-morbidities were mild, mainly represented by diabetes and hypertension, contrary to western studies where the majority of patients admitted to the ICU had several serious co-morbidities such as cancer, autoimmune diseases and a history of organ transplants [17,18]. The third hypothesis is the early use, in our study, of anticoagulants and corticosteroids, in particular dexamethasone. Indeed, we started using corticosteroids in our ICU patients in the first weeks of the pandemic long before the WHO declared their use as a mean of reducing mortality in COVID patients in the ICU [19]. The multiple logistic regression analysis shows that, only ARDS, Brescia score greater than or equal to 2 and ICU admission time >14 days were found to be independent predictors of ICU death in our study. These predictors found in our study are different from those found in most of the literature. A literature review of 13 articles including several meta-analyses identified age, male sex, the presence of co-morbidities particularly diabetes, severe obesity, cardiovascular disease and chronic lung disease, and the presence of biological abnormalities as predictors of mortality consistently related to COVID [10,12,15,16].

All the articles included in this literature review made reference to studies carried out in Western countries, no African studies were included. In our study, some of these factors such as age, male sex, presence of co-morbidities were only associated with mortality in univariate analysis. Our study thus underlines the non-applicability of data obtained in northern countries to sub-Saharan African countries. Indeed, African epidemiological data are very different from those of northern countries. In our study, a delay of admission to an intensive care unit (ICU) > 14 days was identified as one of the 3 factors predictive of ICU mortality. It multiplied the risk of death there by 6. Our results are similar to those of Dananché *et al*. in France and Yang *et al*. in China who reported a significant association between an admission delay of more than 10 days and ICU mortality [10,15]. Indeed, this long delay could be explained by the fact that the majority of our patients were referred from other health facilities where they had stayed for several days for other diagnoses because of the similarity of COVID symptoms with those of certain infectious pathologies found in tropical areas. In addition, for fear of being stigmatized, people who had symptoms did not report them quickly and only went to the health facilities after several days of suffering from the disease. This long delay in admission led to a delay in treatment, making it easier for complications to set in, often leading to death.

The Brescia score is an algorithm specially designed by Italian teams to quickly compare and summarize the severity of coronavirus patients admitted to the ICU. Its discriminatory power in predicting death was superior to other scores such as the Sequential Organ Failure Assessment (SOFA) and other mortality factors found in the literature [9,11]. In our series, the Brescia score was found to be a mortality factor in multivariate analysis (p=0.004) multiplying the risk of death by 6. Our results corroborate those found by Piva *et al*. who reported in Italy that the Brescia score greater than or equal to 2 was significantly associated with the occurrence of mortality [6]. In fact, a Brescia score greater than or equal to 2 is correlated with greater severity of the patient´s condition, which may lead to death [11]. Acute respiratory distress syndrome is a dreaded condition with a very high mortality rate due to its characteristics of acute pulmonary aggression and hypoxemia. It is one of the most common complications in the ICU in patients with coronavirus disease [20]. In many published studies, ARDS has been frequently found as a mortality factor in the ICU [21,22]. This observation was the same in our study where ARDS was significantly associated with mortality in multivariate analysis (p=0.007) and multiplied the risk of death by 6.33. Our results are similar to those of Zhou F *et al*. in China and Arentz M *et al*. in the USA who found ARDS to be a factor in the occurrence of death [12,13,17]. Such a result would be explained by the link established between the delayed admissions of deceased patients, most of whom were admitted with already signs of respiratory failure with collapsed oxygen saturations rapidly worsening the prognosis. The main limitation of our study is the lack of biological data in the majority of our patients. Indeed, at the beginning of the epidemic, no biological laboratory wanted to carry out the biological examinations for fear of contamination of equipment and personnel. The small size of the population studied could be a factor reducing the statistical power of our study.

## Conclusion

ICU mortality among COVID patients in our study is low, which outweighs the negative predictions made for African countries. Factors such as admission time greater than 14 days, high Brescia score and acute respiratory distress syndrome were found in our study as predictive factors of ICU mortality. These predictive factors are only preliminary results but could be an important tool for better management of patients and resources. The difference with the factors identified in developed countries supports the interest of carrying out a large scale study which could allow a better identification of mortality factors in sub-Saharan Africa.

### What is known about this topic

- Age, gender and co-morbidities such as hypertension, diabetes, cardiovascular disease, chronic respiratory disease and cancer are risk factors for mortality from COVID-19 in Europe, Asia and America;
- The mortality rate for COVID-19 is high among patients in intensive care units in occidental countries.

### What this study adds

- The mortality rate of patients infected with COVID-19 was 25% in the intensive care unit of Donka Hospital in Guinea;
- Factors such as the time of admission to the intensive care unit being more than 14 days since the onset of symptoms, the Brescia score ≥ 2 and acute respiratory distress syndrome were the predictors of mortality among patients with COVID-19 in the intensive care unit.
